# Targeting CD200 in Breast Cancer: Opportunities and Challenges in Immunotherapeutic Strategies

**DOI:** 10.3390/ijms26010115

**Published:** 2024-12-26

**Authors:** Sihyang Baek, Kui Cui

**Affiliations:** 1 Western Reserve Academy, Hudson, OH 44236, USA; christinesbaek@gmail.com; 2 Vascular Biology Program, Department of Surgery, Boston Children’s Hospital, Harvard Medical School, Boston, MA 02115, USA

**Keywords:** CD200, CD200R, breast cancer, immune evasion

## Abstract

One of the key factors that contribute to tumor progression and resistance is the immunosuppressive microenvironment of the tumor. CD200 is a recently identified cell surface glycoprotein recognized as an important molecule in breast cancer for its versatile modulation of the immune response via its receptor, CD200R. The interaction between CD200 and CD200R suppresses the immune activities against tumor cells and allows them to be undetected and, in doing so, to escape from the destructive capability of the immune cells. Here, we review recent advances and future trends in CD200-targeted therapies for cancer treatments. We also discuss molecular pathways that include variable expressions across different cancer types and their importance in treatment options.

## 1. Introduction

Breast cancer is one of the most prevalent cancers and a leading cause of death among women. The search for new therapeutic targets and treatment strategies is urgently needed. Central to this interest is CD200, a cell surface glycoprotein involved in the negative regulation of immune responses [1,2]. CD200 binds to its cognitive receptor, CD200R, and the CD200/CD200R interactions regulate the immune tolerance and weaken antitumor immunity within the tumor microenvironment [3]. CD200 expressed in the tumor cell surface interacts with CD200R present in the membrane of various immune cells, and this molecular interaction provides an immune status capable of allowing tumor development and metastasis, while protecting the cancer cells from immune damage [4,5]. CD200 is expressed by many cell types, including tumor cells. Studies show that CD200 inhibits myeloid immune cell activities [6,7]. Specifically, CD200 has been associated with pathologic and prognostic outcomes in breast cancers [8]. For example, its expression differs significantly among breast cancer subtypes and influences its tumor behavior and treatment responses. This expression variation in different subtypes is a chief complication in clinical management, but a good potential therapeutic target [6,9]. Initial clinical trials are found to be promising based on the cumulative experimental and clinical evidence pointing to the potential of therapies targeting CD200 to enhance current chemotherapy and/or immunotherapy strategies [10]. However, we need a good understanding of the tumor microenvironment and its regulation mechanisms, considering the complex repertoire of molecular interactions between the tumor and the immune system of the microenvironment, as well as the opposing roles of CD200 in both tumor growth suppression and promotion [1]. Moreover, genetic and epigenetic variations in the therapeutic targeting of CD200 contribute to the variability in patient response [11]. In this review, we will discuss the immune actions, functional modes, and therapeutic developments of CD200 in the context of breast cancer, with a special emphasis on recent advances in molecular biology, clinical research and therapeutic innovations.

## 2. CD200 Introduction to Cancer Biology

**Figure 1:**
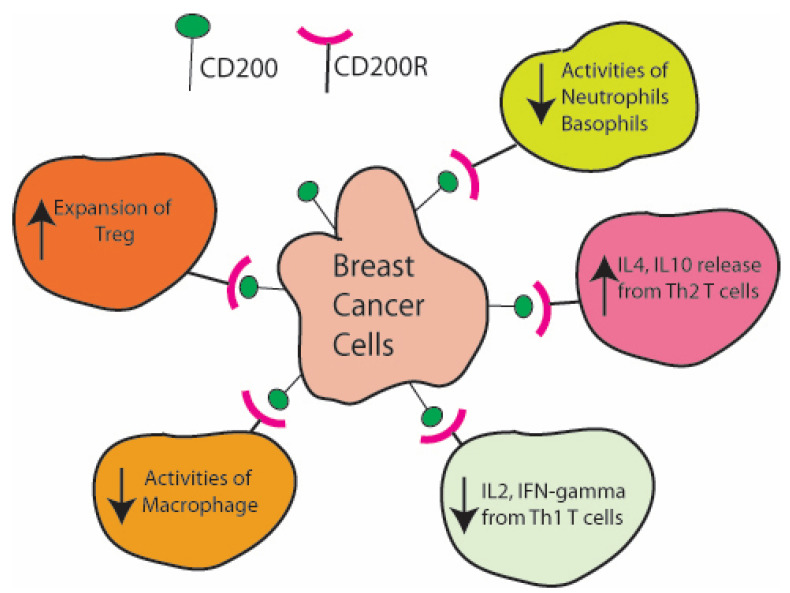
CD200 suppresses the antitumor activities by regulating various aspects of immune functions. CD200 protein expressed on the surface of breast cancer cells instructs various cell types to regulate the immune activities in the tumor microenvironement.

Extensive research has found the critical role of CD200 in the tumor microenvironment through the regulation of the functions of various immune cells [12,13]. The biochemical and molecular interactions between CD200/CD200R have become a point of intense research interest as its blockade, especially with specialized antibodies or peptide blockers, could potentially improve antitumor immune responses [14]. In contrast, the expression of CD200R is limited in limited tissue types and thus, the engagement of CD200 can induce diverse functional consequences [15]. The interaction of CD200R-expressing myeloid cells with CD200 downregulates their activation, promotes an immunosuppressive environment, and suppresses macrophage-driven immune activation [16]. The key target for this interaction is tumor-associated myeloid cells, thus inducing a critical role in cancer progression [17]. These interactions generally inhibit immune cells that would attack the tumor cells otherwise [18]. One mechanism for this immune suppression is that the expression of CD200 on tumor cells enhances the release of IL-10 by tumor-associated myeloid cells, contributes to immune suppression and favors tumor progression and recurrence [19]. For example, Table 1 outlines the expression of CD200 across different cancer types, considering the complexity of the tissue environment, genetic/epigenetic factors and their clinical outcomes. The expression of CD200 has been associated with the characteristics of cancer stem cells shown in head and neck squamous cell carcinomas, and also with the reduced chemosensitivity and radiosensitivity of these cancers [20]. In cutaneous squamous cell carcinoma, the high expression of CD200 has been associated with a poor prognosis [21]. The comparative studies on CD200 expression patterns in cancers are quite unique and deserve mechanistic attention. In breast cancer, where it is generally overexpressed, it is linked to reduced primary tumor growth, while remaining associated with increased metastasis [6]. This overexpression further enhances the levels of its receptor, CD200R, and is linked with higher visceral metastasis to organs like the liver and lungs [22]. Thus, it indicates the role of CD200 in a dual manner for the progression and metastasis of breast cancers. Furthermore, it may upregulate the expression of CD200R and contribute to immune suppression rather than increasing tumor-infiltrating cytotoxic T cells. Indirectly, CD200 promotes tumor growth by making the environment favorable for tumor development [23]. The presence of CD200 on the surface of tumor cells directly inhibits the immune system’s attack on the tumor cells and allows cancerous cells to escape detection [4]. Consequently, one of the promising ways of combating CD200-expressing cancers is through targeting CD200/CD200R interactions [15]. In addition, CD200 suppresses the activation of neutrophils and basophils by promoting the production of IL4 and IL10 proteins from Th2 T cells, and it also reduces IL2 and IFN-gamma secretion from Th1 T cells [3]. Additionally, it affects the function of Treg and macrophages (Figure 1). Notably, the shedding of the extracellular domain (soluble CD200) from basal cell carcinoma represents a novel mechanism of immune evasion that acts against the efficacy of available T-cell immune blockers used to treat these cancers. This interaction provides a potential anti-CD200 therapy or other immune checkpoints in developing immunotherapy against cancer [1]. For example, the local adenoviral delivery of soluble CD200R-Ig molecules enhances antitumor immunity by a blockade in CD200-mediated M2 macrophage polarization along with the increased expression of immunity-related genes [17]. Multiple CD200 splice variants have been reported [24]. A truncated, splice variant form of the full-length CD200, namely CD200tr, functions as a physiologic antagonist of CD200 and inhibits the soluble CD200-induced immunosuppression [25]. Remarkably, this alternative splicing of CD200 is regulated by an exonic splicing enhancer (ESE) located in exon 2 of CD200, which is a putative binding site for a splicing regulatory protein SF2/ASF [26]. The inhibition of SF2/ASF expression resulted in the same splicing pattern as seen upon the deletion of the ESE, while the SF2/ASF overexpression increased the expression of the full-length CD200. Certain human carcinoma tissues express not only full-length CD200 (CD200L) but also its truncated form, CD200S (=CD200tr). Notably, synthetic peptides in the N-terminal of CD200 have been reported to antagonize the immunosuppressive function of the natural CD200 molecule [27,28]. When transplanted into the forebrains of newborn Wistar rats, glioma cells expressing the CD200S form showed a prolonged survival than the same parental cells expressing the CD200L form [29]. Tumor-associated macrophages (TAMs) in the CD200S-expressing tumors displayed dendritic cell (DC)-like phenotypes, suggesting that CD200S may have its own specific effects on tumor immunity independently of CD200L [29].

## 3. Expression of CD200 in Breast Cancer

The difference in the CD200 expression between normal tissues and tumor-invaded tissues is significant and strongly influence the dynamics of the tumor development [23]. In breast cancer, a high expression of CD200 is associated with enhanced tumor growth, although the effects on metastasis are inconsistent [36]. The available data suggest that the role of CD200 in metastasis is not fully understood and somewhat contradictory, with some studies suggesting that it might decrease the risk, and others hinting that it could promote metastasis [5]. Thus, more studies will be needed to fully understand the function of CD200 in modulating disease aggressiveness [5,37]. Serum levels of soluble CD200 proteins have the potential to become a diagnostic tool for breast cancer, and the level of CD200R may offer some idea of the stages of the disease, but these studies still require additional confirmation and validation [5,38,39]. Obviously, changes in the level of CD200 depend on tissue type and their value in diagnosis and prediction, as has been emphasized in several studies. A high heterogeneity has also been found in the expression of CD200 between different types of breast cancer [40]. For example, the expression of CD200 expression may vary, such as in basal-like breast cancers, which lack estrogen receptors and HER2 but express basal cytokeratin, HER1, or cKIT [37]. This variation has been seen to relate to certain biological behaviors and differential treatment responses among the various subtypes of breast cancers [41] (Table 2). Further, the role of CD200 in metastasis in breast cancers was demonstrated in studies showing that the neutralization of CD200 by monoclonal anti-CD200 antibodies decreases tumor metastases and was associated with increased cytotoxic antitumor immune cell flux into the regional lymph nodes [36]. CD200 levels may, thus, be one additional factor in defining metastatic risk, perhaps differently for the various subtypes of breast cancer. However, there are several similarities in the behavior of CD200 in breast cancer and other cancers, even though it seems to act somewhat differently.

Genetic factors also influence the level of CD200 and impact tumor growth and dissemination [5]. They can affect CD200 regulation in breast cancer and control its role in tumor growth [6]. CD200 expression is also found in acute myeloid leukemia stem cells, including those carrying NPM1 mutations [52]. The co-expression of CD200 with some stem cell markers points to a possible route through which tumors may bypass the host’s immune surveillance system in an epigenetically controlled manner. CD200 expression in head and neck squamous cell carcinomas is related to features typical of cancer stem cells [20]. It is associated with a poor response to chemotherapy and radiation and suggests that epigenetic factors controlling CD200 expression could be associated with treatment resistance. The overexpression of CD200 in breast cancer is associated with increased tumorgenicity, metastasis and poor prognosis [33]. Therefore, CD200 has emerged as a potential marker for a dismal prognosis. High CD200 levels in breast cancer cells may also impact immune cells in lymph nodes and be related to lymph node status [3].

These observations suggest that the overexpression of CD200 could be one of the reasons for poor prognosis in breast cancer [6]. As the high expression of CD200 in cutaneous squamous cell carcinoma is related to immune evasion, it is considered a potential biomarker predictive of reduced overall survival and may imply similar roles in breast cancer. The expression of CD200 in head and neck squamous cell carcinoma was associated with characteristics similar to cancer stem cells and with the resistance to chemotherapy and radiation [20]. Similar effects from such therapies may occur in breast cancer. Monoclonal antibodies directed against CD200 have shown the ability to inhibit metastatic dissemination and increase cytotoxic antitumor immune cells in lymph nodes in preclinical models [1]. This suggests that CD200 could be important in metastasis and immune escape in breast cancer, although further validation is needed. CD200 and its receptor, CD200R1, are under investigation as diagnostic and prognostic markers in this malignancy [22]. Most studies have confirmed that the overexpression of CD200 is an important marker of poor prognosis. Among the important effects of CD200 expression by breast cancer cells are those on the interactions with immune cells in the microenvironment [6]. CD200 expression may support tumor growth and modify the composition of tumor-draining lymph nodes, one of the important components, locally, of any immune landscape [3]. CD200 is an inhibitory molecule that dampens the antitumor immune function, a potential target for immune checkpoint therapy in breast cancer [1]. CD200 promotes an immunosuppressive environment and contributes to tumor immune escape [53]. CD200 in tumor cells suppress antitumor responses by facilitating immune evasion [54]. Anti-CD200 treatments can be useful in cancers where high levels of CD200 are observed [1]. It also influences the activity of other immune cells, such as T cells and macrophages [18]. The blockade of CD200, in preclinical models, by anti-CD200 monoclonal antibodies was demonstrated to reduce tumor metastasis and increase cytotoxic antitumor immune cells in lymph nodes. This showed that CD200 plays a part in regulating the invasion of immune cells into the tumor microenvironment and their further activity.

## 4. CD200 and Immune Evasion

Immune evasion includes the protection of tumor cells from immune attacks and enables the growth and dissemination of cancer. In breast cancer, this occurs through antigen presentation, the production of immunosuppressive factors, and prompting poor immune reactions within the tumor territories [55]. CD200 expression has been implicated in helping tumors evade immune responses by binding to its receptors on immune cells, such as natural killer cells and macrophages, leading to the CD200R-mediated suppression of immune activity against tumor cells and the consequential growth and metastasis of tumor cells [4,12,37,56]. Studies confirmed the roles of CD200 in immune escape during the course of breast cancer [1]. The blocking of its interaction with CD200R on the surface of immune cells can reduce tumor spread, enhance immune responses, and offer a new avenue of anti-cancer therapies [14]. The capability of CD200 was further recorded in other malignancies, such as melanoma, renal cell carcinoma and ovarian tumors, using anti-CD200 antibodies, which were shown to initiate vigorous immune responses against breast cancer and other solid tumor types [54]. On the other hand, CD200 was reported to trigger the induction of the immune tolerance, which could be hijacked, in immunotherapy, to attack cancer cells [15]. Thus, this paradigm has found CD200 to be a promising treatment target and also a biomarker across diverse types of cancers, given that high levels of this protein are associated with tumor growth. Notably, blocking CD200 could enhance antitumor immunity and contribute to stronger immune responses in tumors [17]. The overexpression of CD200 in breast cancer cells is associated with accelerated tumor growth and impairs prognoses. Similarly, the inhibition of CD200 reduces tumor growth in syngeneic mouse models [6]. Thus, the CD200-targeting therapies may better be combined with other treatment modes to attain the maximal benefits for breast cancer patients [57]. Together, targeting CD200 using antibodies or peptide blockers presents a promising area of study and a potential future mode of treatment. This represents the potential of CD200 as a therapeutic target in the treatment of breast cancer and other cancers. (Outlined in Table 3).

## 5. Targeting CD200 for Immunotherapeutic Intervention of Breast Cancer

Currently, antibodies targeting CD200 and CD200R are being developed for the purpose of the immunotherapy treatment of breast cancers [18]. Studies have demonstrated that blocking the molecular and biochemical interactions of CD200 and CD200R indeed reversed the immune-suppressing effects [2]. Monoclonal antibodies and synthetic peptides are currently being tested to reduce the suppressive effects of CD200 on the immune system [59]. The role of CD200 in cancer immunotherapy could augment the immune system and modulate its typical function toward the tumor antigens [60]. In fact, early research showed that monoclonal antibodies had the potential to combat breast cancer progression by breaking down the tumor-supporting microenvironment. Conjugated with a peptidic inhibitor, CD200 proteins target important pathways to decrease the immune suppression of the tumor and amplify immune activity [10]. The treatment of other diseases, such as non-Hodgkin’s lymphoma and chronic lymphocytic leukemia, using these blocking antibodies has yielded quite promising results [61]. Indeed, the success of monoclonal antibodies in cancer therapy warrants their application as treatment options against breast cancers [18]. On the other hand, CD200 neutralization provoked a profound humoral immune response against tumors expressing this molecule, including mammary tumors, and its combination with other therapies could offer a more robust antitumor activity [1]. Further studies and the application of immune-modulating strategies will be needed to fully develop CD200 as an outstanding therapeutic target in cancer therapy.

Moreover, vaccines targeting CD200 have been shown to increase, in preclinical studies, the immune responses against CD200-expressing tumor cells, improve immune detection, and effectively killing the cancer cells [11,62,63]. The preclinical vaccination against CD200 has been shown to enhance antitumor activity and inhibit blood vessel growth. This reduces tumor progression in models of primary and metastatic cancer and offers a potential relevance for breast cancer [64,65,66]. Such success warrants the extension of comparable approaches against CD200. The tumor environment, including mechanisms associated with CD200, may determine the extent of immune suppression occurring from vaccines in preclinical studies. Immune suppression weakens or abrogates responses [10]. The future of cancer vaccines targeting CD 200 is bright. In addition, combination treatments involving chemotherapy or other forms of immunotherapy along with anti-CD200 approaches are also under development. These treatments aim to achieve better outcomes by attacking, in various ways, the growth of cancer and its evasion from the immune system. With this, treatment outcomes improve significantly and, additionally, may enhance the survival and control of disease [67,68].

Recent studies have identified more than a dozen different combinations of methods to help improve immunotherapy. The combination of CD200 therapies with PD-1/PD-L1 and CTLA-4 inhibitors are being studied actively, and early results suggest a promise for increased therapeutic effects. The immune-modifying role of CD200 therapies supports their potential to improve outcomes [67,68]. The combination of CD200 therapies with CDK4/6 inhibitors also enhances the effectiveness of hormone therapy in hormone receptor-positive breast cancers. This strategic combination modulates the cell cycle at key checkpoints, potentially addressing immune evasion tactics and offering additional major treatment options [68]. With the combination of CD200-targeted therapies and the endocrine treatment of hormone receptor-positive, the treatment of HER2-negative breast cancers is expected to be promising. Such treatments can target various aspects of hormonal shifts and immune evasion [69]. Thus, CD200-targeted therapies may greatly enhance the treatment of breast cancers by offering a multi-target approach that may alter the course of current treatments. Table 4 summarizes the clinical trial results for CD200-targeted therapies against breast cancer.

The ongoing anti-CD200 trials are mainly focused on proving the efficacy and safety of these treatments in patients with breast cancers. Most treatments are being investigated in combination with CDK4/6 inhibitors. This examines complementary mechanisms since anti-CD200 therapies deplete immunosuppressive signals within the tumor microenvironment and amplify immune-mediated tumor attacks, while CDK4/6 inhibitors block tumor proliferation via the induction of G1 cell cycle arrest [1]. While the direct interactions between CD200 and CDK4/6 pathways are not well documented, the preclinical evidence suggests that the immune modulation from anti-CD200 might potentiate the effects of CDK4/6 inhibitors in hormone receptor-positive, HER2-negative breast cancers [18]. This dual approach shows great promise in improving clinical outcomes through the inhibition of tumor growth and enhanced immune responses [77]. The outcomes from these trials are expected to contribute to developing better therapeutic strategies for cases with hormone receptor-positive and HER2-negative, with a possibility of improvement in the clinical prognosis using personalized treatment plans [78]. The HER2 gene has been targeted, in early clinical trials, for tumor reduction and safety. Indeed, these studies confirmed the efficacy and safety of this treatment by reducing the tumor size in most cases [74]. Moreover, preclinical studies using CD200-targeted therapies against aggressive brain tumors, such as glioblastomas, also exhibited a great deal of promise [10]. The controversial involvement of CD200 in tumor metastasis, its differential expression, and its heterogeneous impact on tumor growth indeed complicate its targeting in breast cancer. Such opposing effects require advanced therapeutic strategies if the best results of the therapies for patients are yet to be completely defined [5]. Effective targeted therapies for CD200 thus appear to be challenging because CD200 expression varies widely across different tumor types, including breast cancers. For this reason, treatments must be personalized for each patient, which limits the wider use of this therapy [6,59]. Furthermore, blocking CD200 can cause various reactions and requires careful patient selection and monitoring for non-trivial side effects. Focused treatments are still needed due to these challenges [15]. One new approach uses a peptide inhibitor to block CD200 receptors, along with another peptide that activates the receptor. This treatment plan has proven to be effective against induced tumor immunosuppression and extended the immune responses specific to tumor antigens [10]. Other novel combined immune therapies targeting myeloid cells and checkpoint inhibitors are also under active development, and are intended to overcome the resistance commonly exhibited by targeted therapies [79]. Advanced drug delivery systems, like CD-targeted gold nanoparticles, show promise in stopping metastasis [80]. Together, targeting CD200/CD200R is regarded as a promising approach in cancer immunotherapy, although its ability to prevent tumor growth and spread is not fully understood [13].

## 6. Clinical Trials and Results

CD200 therapy in breast cancer could only be developed through clinical trials, which need to consider not only efficacy, but also safety and integration. A key focus for such trials is the degree to which these CD200-targeted therapies enhance antitumor immunity and how they might be combined with other combining treatments, such as chemotherapy or other immune therapies [2,81]. Several investigations have pursued specific agents, including peptide inhibitors targeting CD200 that have already shown a promise in early tests [10]. The safety and effectiveness of vaccines targeting CD200 in breast cancer were also tested [37,38,82,83]. These findings emphasize the use of CD200-targeting therapies only toward correctly identified patients. These may also require additional treatment plans for better outcomes in breast cancer. While the blockade of CD200 can enhance the immune responses against tumors, it might also promote inflammation and may, therefore, boost the growth of inflammation-driven cancers [15]. Preliminary studies highlighted that CD200-targeted therapies have potential as immunomodulatory drugs that show promise, especially in CNS-related cancers; this principle may even be applicable to breast cancer [14]. Moreover, the role of CD200 in immune evasion and treatment resistance in pancreatic cancer hints at its potential effects as a biomarker for analogous therapeutic strategies in breast cancer, covering pre- and post-surgery interventions [84]. Diagnostic tests for their detection could be developed based on the outcome of current clinical studies. These studies and clinical trials will likely help us better understand the complicated aspects of CD200-targeting treatments and will present new opportunities to improve antitumor responses [85]. However, these methods still face challenges and need broader approaches to treat breast cancer more effectively.

## 7. Complexities and Challenges of CD200-Targeted Breast Cancer Therapy

Breast cancer cells take advantage of the complicated molecular interactions within their microenvironment to evade the immune system and acquire resistance against CD200 therapies [86]. A few major mechanisms of drug resistance were recently identified to obscure the effectiveness of such therapies [87]. While the overexpression of anti-cell death proteins, such as BAG3, and other pathways protective against cell damage confer cancer resistance, their contribution to the outcome of CD200-targeted therapies against breast cancer remains unclear [88]. In susceptible cases, the co-chaperone, anti-apoptotic BAG3 revealed a high expression in breast cancer cells that were resistant against a certain class of chemotherapy drugs [89]. Its overexpression was linked to the enhanced resistance against the action of drugs and lesser response to signals of cell death [88]. The resistance from the breast cancer stem cells makes the treatment more complicated since these cells can lead to recurrence and metastasis [90,91]. This issue is further complicated by long non-coding RNAs (lncRNAs) that manage cell functions. The involvement of lncRNAs, including MIR200CHG, in tumor behavior and resistance is extensive, although their connection to CD200-targeted therapies is still unclear [92]. lncRNA acts through the interaction with the transcription factor Y-box binding protein 1, maintains its stability and is important in the gene expression associated with tumor growth, apoptosis, invasion and drug resistance [92]. These interactions provide the malignant properties of the cancer cells [92,93]. Clinical observation and case studies have presented the complex causes of the resistance mechanisms. The resistance of the CDK4/6 inhibitors in breast cancer patients is characterized by specific and general cell processes that pose efficient barriers against treatments that would help improve the survival of patients with the disease [94,95]. This heterogeneity, in the case of breast cancer, shows the varied responses of patients to therapies based on genetic and immune factors [96,97]. These differences thus build up the heterogeneity observed within the treatment outcomes of the overall patient population [98]. These may be dictated by certain genetic mutations or gene variations that could affect the expression of both CD200 and its receptor, CD200R, on the tumor and immune cells [99]. Genetic diversity can thus alter the effectiveness of the immune checkpoint blocker and may reflect variable treatment responses among patients [12,81].

Other genomic variations influence the body’s processing of and response to therapeutic agents in drug-metabolic and transport pathways, which moderates the effectiveness and toxicity profiles of such agents [100,101]. It is thus crucial for the success of a given therapy. Epigenetic modifications regulate the responsiveness to treatments [102]. These epigenetic modifications may change the expression of genes in the CD200–CD200R axis and regulate the general immune response [18]. In fact, the therapy-induced alterations of the tumor microenvironment can enhance immune responses or heightened immunosuppression. There is also a great heterogeneity within treatment outcomes not only across but also within patient groups [103]. They contribute to the disparity in immune responses and further give rise to the disparity in the status of the immune cells of the tumor microenvironment [104]. However, the overall composition and function may be dissimilar in immune cells, especially in different types of immune cells. These dissimilarities arise between distinct individuals, and even within patient groups with the same diagnosis [105]. Indeed, such variations can affect immune suppression levels or activation caused by therapy, which support an individualized approach to the implementation of CD200-targeted therapies [59]. By understanding each patient’s own tumor and immune system interactions, personalized treatment can be accomplished with the use of CD200-targeted strategies [106].

Generally, it is not easy to target breast cancer cells without disturbing the presence of normal cells [21,107,108]. The challenge is to target CD200-expressing cancer cells without harming healthy tissues. Therefore, there is an urgent need for approaches that limit the off-target effects to enhance patient safety [15]. One of the major challenges involves the delivery of such therapeutics against CD200 into the tumor microenvironment. Other challenges are maintaining stability, ensuring bioavailability in the system and overcoming the physical and biological obstacles presented by the tumor microenvironment to achieve adequate levels at the tumor site [109]. Drug targets and pathway activation may shift, or drugs may be metabolized differently, in a way that the effectiveness of therapies targeting CD200 is lost gradually. CD200-targeting immunotherapies are associated with several immune-related adverse events and complicate patient care and safety management [18]. These side effects should be managed to ensure that the treatments can be given safely. The therapies targeting CD200/CD200R block or interrupt immunosuppressive signals mediated by CD200 on tumor cells and augment immune attack against tumors [1]. This may disturb the regulation of immunity and cause overactive immune responses known as Immune-Related Adverse Events (IRAES) [110]. The major IRAEs of CD200-targeting immunotherapies are mainly autoimmune toxicities that complicate patient care and the management of safety [111]. Examples of such commonly observed toxicities include colitis, pneumonitis, and hepatitis. Grade 3–4 IRAEs occur more frequently with CTLA-4 inhibitors at 31%, compared to 10% for PD-1 inhibitors [111]. Common gastrointestinal events, such as colitis, occur in approximately 5.3% of the patients who are receiving anti-CTLA-4 therapies [112], whereas diarrhea may be seen in up to 14.5% of patients in combination therapies [113]. Other notable endocrine IRAEs include hypothyroidism and hypophysitis. Hypothyroidism is more often related to PD-1 inhibitors, with an OR of 4.3 (95% CI 2.9–6.3), while hypophysitis is associated with CTLA-4 inhibitors, with an OR of 6.5 (95% CI 3.0–14.3) [111]. However, severe grade 3–4 endocrine toxicities are less common [112]. The management strategies for IRAEs include corticosteroids and immunosuppressants, such as infliximab for refractory colitis [112]. Early symptoms, such as diarrhea and fatigue, have to be promptly identified to minimize risks and keep the treatment going [113]. A team approach is needed to manage the side effects of CD200-targeted therapy [114]. The symptoms of IRAE need close monitoring and patients should be educated regarding early signs [115]. Also, physicians must use immune-suppressing drugs quickly. Treatment might need to be temporarily or permanently stopped in the case of severe side effects. In fact, the biomarkers for IRAE are critical, and for these therapies targeting CD200 to be safe and effective in breast cancers, more clinical data accumulation will require further clarification on how those risks could be reduced [116].

## 8. Future Research Directions and Research Avenues

Targeting immune checkpoint CD200 in breast cancer is challenging but shows great promise [18]. Complex changes in both the immune system and different tumor environments, besides genetic variability, call for new strategies to enhance existing treatments [14]. Recent investigations have demonstrated a link between the gut microbiome and the outcome of immunotherapy; however, how the microbiome influences CD200 expression remains unknown [117]. Future studies should examine the influence of the microbiome on CD200 expression by both tumor and immune cells during human breast cancer. This may reveal new approaches through which this pathway could be manipulated to improve the effectiveness of CD200-targeting therapeutic approaches. Additionally, identifying specific bacterial species that impact the expression of CD200 could allow for the development of probiotics with positive effects on immune function [118,119,120]. Advanced computer modeling to predict patient outcomes with CD200-targeted therapies could significantly enhance personalized treatment in breast cancer [119,121]. The models need to be designed with complete data from genetic, protein and clinical studies—each proposed for the accurate forecast of the treatment outcome [122]. To this end, studies should be directed to develop algorithms that identify patterns of either treatment response or resistance [123]. This will help doctors personalize the treatments, make therapies more effective and decrease the exposure to ineffective treatments. This involves long-term clinical studies that track changes in CD200 expression throughout cancer treatment into remission. A better understanding of these processes may ultimately offer flexible treatment strategies where treatments targeting CD200 are varied over time to correspond with the changes in activity patterns of the molecule. A combination of CD200 targeting with novel immunotherapeutic approaches, including CAR T cells and bispecific antibodies, presents new opportunities in tumor treatment [124]. Future studies should be directed to develop inhibitors that could block the interaction of CD200 with its receptor and, at the same time, be able to deliver a cytotoxic drug directly to the tumor itself [123]. Such dual-action inhibitors will be much more efficient in destroying tumor cells, diminishing immune suppression, thereby yielding better treatment results.

## 9. Conclusions

Targeting therapy through the use of CD200 represents one of the new approaches to manage breast cancer and may contradict the immune escape mechanisms developed by cancer cells. Blocking its activity allows treatments that enhance immune function. Preliminary studies show that blocking CD200 improves the immune response and delays tumor growth. In this regard, further development will be necessary to promote the precision of inhibitors of CD200, comprehensively understand the resistance to them and develop combination treatment strategies that appropriately exploit such knowledge. Treatments targeting CD200 thus provide a promising opportunity to improve outcomes in the management of breast cancer, although additional confirmation in clinical trials is needed. These advances should lead to more effective and precise strategies and make CD200-targeted treatments a standard part of the treatment options for advanced breast cancer.

## Figures and Tables

**Table 1:** Comparative insights: CD200 in breast cancer vs. other cancers.

Cancer Type	CD200 Expression	Immune Context	Genetic/Epigenetic Influences	Clinical Outcomes	Reference
Breast Cancer	Variable; Inversely related to primary tumor growth and positively related in some subtypes associated with metastasis.	Influenced by immune competency of the model: immunocompetent versus immunocompromised.	Expression influenced by subtype-specific genetic and epigenetic modifications.	Differential: suppressive in primary tumors but may promote metastasis in aggressive cases.	[1,4,5,18]
Head and Neck Squamous Cell Carcinoma	High; correlates with cancer stem cell features and treatment resistance.	Effectiveness possibly reduced in immune privileged sites.	Associated with resistance to chemoradiation.	Generally promotes tumor progression and resistance to treatment.	[20,23,30]
Melanoma	Overexpression inhibits tumorigenesis and metastasis.	Mechanism dependent on the interaction with myeloid cells within the immune system.	Less clearly defined but most likely involving regulatory networks affecting immune suppression.	Suppression: Limits tumor progression and metastasis.	[11,13,31]
Cutaneous Squamous Cell Carcinoma	High; independent prognostic factor for poor outcomes.	Acts through immune cells, for example, in the skin microenvironment.	Less well defined, but is postulated to utilize comparable pathways to that seen in other epithelial tumors.	Sustains tumor development and worse prognosis.	[21,32]
Multiple Myeloma and Acute Myeloid Leukemia	Expressed in Multiple Myeloma and Acute Myeloid Leukemia; associated with immune evasion.	Impact on immune suppression mainly within the bone marrow microenvironment.	Involvement of mutations affecting interactions with immune cells.	Correlated with poor prognosis due to increased immune evasion.	[33,34,35]

**Table 2:** CD200 in various breast cancer subtypes.

Cancer Type	CD200 Expression Levels	Immune Context	Genetic/Epigenetic Factors	Clinical Implications	Reference
Luminal A	Moderate to High	Moderately suppressive immune microenvironment; CD200 expression is associated with resistance to hormone therapy.	Epigenetic regulation impinges on hormone receptor interaction.	May be a marker for hormone therapy resistance; less implicated in metastasis.	[1,42,43,44]
Luminal B	Moderate to High	More aggressive as compared to Luminal A. It is characterized with a moderate immune suppression feature associated with CD200.	Higher proliferation rate due to interference by genetic pathways involving even CD200.	Resistance for treatment; maybe a combined immunotherapy target.	[43,44,45,46]
HER2-Enriched	High	Strong immune evasion overexpression of CD200 associated; linked to resistance against HER2-targeted therapy.	Overexpression linked to the signaling pathways of HER2.	Poor prognosis marker, promising for HER2-targeted therapies in combination with inhibitors targeting the CD200.	[47,48,49]
Triple-Negative Breast Cancer (TNBC)	Variable (high expression in basal-like subtype)	High heterogeneity in immune response; CD200 linked to immune suppression and metastasis.	Epigenetic drivers orchestrate CD200 in the basal-like subtype of TNBC, dictating chemosensitivity.	Generally associated with poor prognosis, though therapy response varies across subtypes.	[50,51]

**Table 3:** Mechanisms of CD200-mediated immune evasion and immunotherapy targets in breast cancer.

Mechanism/Feature	Description and Impact on Immune Evasion	References
CD200 with CD200R Interaction	Suppresses immune responses that would otherwise be stimulated to target and destroy tumor cells. Suppression of the immune response is antagonized upon treatment with anti-CD200 antibodies, and tumor growth is significantly reduced through the reactivation of immune cell functions. The interaction between CD200 and CD200R downregulates immune responses through multiple cell types due to the suppression of T-cell activation and proliferation. It results in impaired cytotoxic activities of T cells, dysfunction, and apoptosis of NK cells, while modulating the activity of dendritic cells and macrophages that promotes the immunosuppressive environment within the tumor.	[1,15]
Immunosuppressive Microenvironment	The interaction between CD200 and myeloid-derived immune suppressive cells preserves tumor immunosuppressive function. Through this mechanism, CD200 has also become a target for immune checkpoint inhibitors aimed at specific immune restoration for the abrogation of tumor-mediated immunosuppression.	[7,18]
Interaction with Tumor-Associated Macrophages	CD200 may potentially activate tumor-associated macrophages to dendritic cell-like antigen-presenting cells, thereby activating CD8+ cytotoxic T lymphocytes and inducing the apoptotic elimination of tumor cells.	[17]
Overexpression in Malignancies	CD200 is overexpressed in the membrane overexpressed in certain hematological malignancies or solid tumors that lead to immune suppression and natural hosts antitime or immune failure.	[14,54]
Metastatic Capacity Augmented	The role of CD200 in augmenting metastatic capacity in squamous cell carcinoma involves the active inhibition of myeloid cell activation and the interaction with CD200R+ immune cells.	[15,16]
Potential for Overcoming Immunosuppression	The treatment with CD200 inhibitors significantly improved survival among vaccinated glioma- and breast carcinoma-bearing mice, suggesting a potential overcoming of tumor-induced immunosuppression.	[10,58]
CD200 Blockade	A blockade of CD200/CD200R could enhance the immune activity against the tumor and may even prevent tumor growth.	[3]

**Table 4:** Clinical trial results for CD200-targeted mono- or combinational therapies in breast cancer.

Category	Subcategory	Details	References
Efficacy Data	CDK4/6 Inhibitors	Pre-clinical and clinical trials on CDK4/6 inhibitors have shown significant improvement in progression-free survival for hormone receptor-positive HER 2 negative advanced breast cancers. Palbociclib, a specific CDK4/6 inhibitor, has been approved for advanced hormone receptor-positive, HER2-negative breast cancer treatment in combination with letrozole or fulvestrant.	[70,71,72,73]
	Suicide Gene Therapy Directed against ErbB-2	ErbB-2-targeted suicide gene therapy entered a Phase I trial in breast cancer and demonstrated targeted gene expression in up to 90% of cases to indicate feasibility and safety.	[74]
Safety and Tolerability	CD200 Blockade	Though effective, CD200 blockade needs careful patient selection and monitoring due to the risk of promoting tumor growth in inflammation-dependent tumors.	[15,75]
	Targeting the CD200/CD200R Pathway	Monoclonal antibodies or peptide inhibitors in this pathway have proven effective in boosting antitumor immune activity; however, the response rates are differential, and some serious immune-related adverse events may occur.	[15,59]
	CD200AR-L Peptide Inhibitor	The inhibition of CD200AR-L by using a CD200AR-L peptide inhibitor significantly improved the median overall survival in dogs with high-grade glioma; it may be effective in breast cancer.	[10]
Clinical Trial Outcomes with CD200-Targeted Therapies	Successes with CDK4/6 Inhibitors	Palbociclib, ribociclib, and abemaciclib demonstrated promising results in trials for the CDK4/6 inhibitors—in particular, for hormone receptor-positive and HER2-negative breast cancer, an improvement in progression-free survival.	[76]
	Immunotherapy Using CD200AR-L and GBM6-AD	CD200AR-L can be used in the treatment for or targeting recurrent glioblastomas, and the therapeutic gain obtained in the treatment can be equally transferred to breast cancer treatment, and this subsequently enhances immune responses that are likely to benefit patients with breast cancer.	[10]
	ErbB-2-Directed Suicide Gene Therapy	A phase I clinical study of erbB-2-directed suicide gene therapy in breast cancer showed targeted gene expression in a significant proportion of cases, indicating that this approach is feasible and safe.	[74]
	Limitations of CD200 Blockade	The antitumor response enhancement characteristic of CD200 blockade can also promote growth in tumors that proliferate under inflammatory conditions, hence the need for careful selection and monitoring of the patients.	[1,15,75]
